# Distal tibial trabecular morphometry in a Sprague Dawley rat model of fetal alcohol syndrome: a micro focus X-ray computed tomography case-control study

**DOI:** 10.11604/pamj.2023.46.35.37151

**Published:** 2023-09-25

**Authors:** Robert Ndou, Nura Kaura Bello, Vaughan Perry, Diana Pillay

**Affiliations:** 1 Department of Anatomy and Histology, School of Medicine, Sefako Makgatho Health Sciences University, Ga-Rankuwa, Pretoria, South Africa,; 2 School of Anatomical Sciences, Faculty of Health Sciences, University of Witwatersrand, Johannesburg, Parktown, South Africa

**Keywords:** Bone trabeculae morphology, tibia, osteoporosis, prenatal alcohol

## Abstract

**Introduction:**

intrauterine alcohol exposure has adverse health effects on the offspring, which may result in fetal Alcohol Spectrum Disorders (FASD). The neurological and craniofacial aspects have been well studied; however, long bones have received limited attention despite the short stature reported in FASD children.

**Methods:**

time-mated (n=13) pregnant Sprague Dawley dams were assigned to either the ethanol (n=5), saline control (n=5) or untreated group (n=3) which received no treatment. The ethanol and saline control dams were treated with 0.015ml/g of 25.2% ethanol or 0.9% saline, respectively. Treatment was for the first 19 days of gestation. Two pups from each dam were used and terminated at 21 days of age. Paired tibiae were harvested. Each bone was scanned using a Nikon XTH 225L 3D-μCT to investigate trabeculae morphometry.

**Results:**

the ethanol group had less bone to total volume (BT/TV), thinnest trabeculae (TbTh) which were less spaced (TbSp) compared to the controls. However, number of trabecular (TbN) remained unaffected in all three groups. Tibial length was similar in all three groups; however, the distal metaphysis volume was smallest in the ethanol group. Logistic regression showed that the distal medullary canal area and trabecular separation were the main parameters affected the most in gestational alcohol. The negative correlation of trabecular thickness and spacing in the ethanol group may be a contributor to bone weakness.

**Conclusion:**

gestational alcohol exposure affects bone internal morphology in addition to the bone size. Overall, this study supports the findings of clinical observation of small stature in FAS children.

## Introduction

Intrauterine alcohol exposure has adverse effects on the long-term health of the offspring, resulting in numerous problems described as Fetal Alcohol Spectrum Disorders (FASD). Fetal alcohol syndrome (FAS), characterized by neurological anomalies, craniofacial dysmorphology and growth retardation is the worst of these disorders [1]. The latter presents with, low body weight, short stature, and low weight to height ratio [2]. Presently, in South Africa, alcohol abuse prenatally is a growing concern. South Africa is reported to have the highest incidence (6%) of FAS worldwide [2]. This high prevalence may be the result of, poverty, socioeconomic status, and lack of education. This syndrome is regarded as a financial and social burden. Children with this condition need special schools and have a higher propensity to fractures and osteoporosis later in life. All these issues add to the burden of this condition, and it overwhelms the health system worldwide. Currently, in South Africa there is no surveillance system to monitor children with this condition thus many are misdiagnosed, which further contributes to the problem of this disease [3]. Gestational alcohol exposure may result in osteoporosis and increased fracture risk in adulthood; therefore, it is worth investigating the effects of prenatal alcohol exposure on bone microarchitecture [4]. Osteoporosis is characterized by a reduction in bone mass and disruption of trabecular and compact bone structure. Alcohol causes a reduction in compact and trabecular bone density and trabecular bone volume in rats [5]. Similarly, Chen *et al*. (2012) found a decrease in trabecular number in the hamster femur following alcohol consumption [6]. This is thought to be due to a decrease in the osteoblast number and size as previously reported in alcohol exposed rats [7]. The rationale for this study is on the premise that, in South Africa, it is common for women to binge-drink in secrecy while attempting to conceal this behavior from their spouses, families, and communities to avoid the public disgrace associated with women drinkers [8] in addition to the associated bone deficiencies. Considering this, the aim of this study was to investigate bone microarchitecture in a rat model that mimics binge-drinking. The specific objective was to obtain tibiae of 3-week-old rat pups from dams that were given alcohol in pregnancy. The other objective was to measure bone length (tibia), and trabecular morphometric parameters from Micro CT data. We propose that gestational alcohol may have a detrimental effect on bone trabecular morphometric parameters at 3 weeks´ postnatal life. This study will provide insight on the long-term effects of prenatal alcohol exposure with respect to bone, microarchitecture. The results may pave the way for development of future therapies to counteract the adverse effects of intrauterine alcohol exposure in postnatal skeletal development.

## Methods

**Study design:** a case-control study which was designed and conducted to investigate the effect of gestational alcohol on the trabecular morphometric parameters of the Sprague Dawley rat tibia.

**Study setting and group allocation:** all study animals were bred and kept at the Central animal services (CAS), University of Witwatersrand, Johannesburg. These animals were kept under pathogen free conditions, temperature-controlled environment (23°C ±2°C) and a 12-hour light/dark cycle. Pregnant dams were individually housed in plastic cages, allowing free movement within. Access to tap water and standard rodent diet was unrestricted. Thirteen female virgin Sprague Dawley rats weighing between 260-350g were used to obtain 26 pups for the study. Dams (n=13) were allocated into either the ethanol (n=5), saline control (n=5) and untreated control groups (n=3). Fewer animals were used in this group (n=3) to reduce the number of animals required for the study. The ethanol and saline groups were treated with 0.015ml/g of either 25.2% ethanol or 9% saline, respectively, through oral gavage for 19 days from the first day of gestation, determined by the presence of a vaginal plug. The untreated control group received neither ethanol nor saline. The dams were allowed to litter naturally, and the pups remained with their dams until the age of 3 weeks before they were terminated by pentobarbital intraperitoneal injection. From each dam, 2 pups were used as follows: ethanol group (n=10), saline controls (n=10) and untreated controls (n=6). Upon termination, bilateral tibia were then individually immersed and stored in 10% buffered formalin to continue fixation until further processing. The sample sizes were chosen to enable adequate statistical analysis.

**Figure 1:**
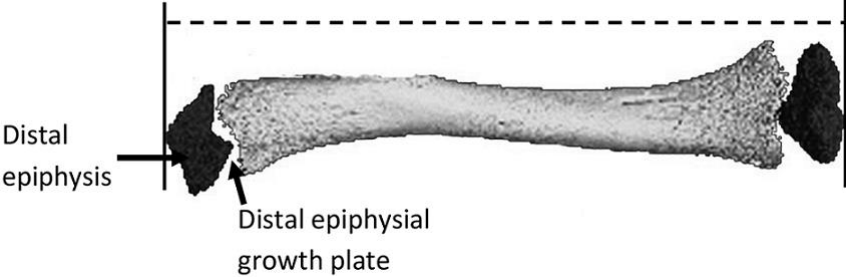
parts of the tibia studied; tibial length

**Variables:** the variables in the study are given in Table 1 and an illustration of the parts of the tibia studied is given in Figure 1.

**Table 1:** trabecular parameters assessed

Parameter	Definition
BAC	Blood alcohol concentration
Bone length	The length from the proximal to the distal end of the tibia in millimeters
Epiphysial volume	The volume of the epiphysis from the the most distal aspect to the distal epiphyseal growth plate
	Represents the ratio of material (bone) volume to total volume
TbTh	Specifies the mean thickness of trabeculae.
TbN	Shows the mean number of trabeculae per unit
TbSp	Indicates the mean distance between trabeculae

TbTh: trabeculae thickness; TbN: trabeculae number: TbSp: trabeculae spacing; BAC; BV/TV: bone to total volume

### Data resource and management

**Determination of blood alcohol levels:** to monitor blood alcohol levels among the pregnant dams, blood from the tail vein was sampled into heparinized capillary tubes weekly, an hour after treatment for the saline control and ethanol dams. All blood samples were centrifuged, and serum was stored at -80ºc until analyzed for BAC (Kit).

**Three-dimensional micro-focus X-ray computed tomography (3D-μCT):** the bones were individually wrapped in Styrofoam and placed in a plastic container. The scanning voltage was 70kv, the X-ray current was 400μa, with a frame averaging of 4 and a resolution of 15μm.

**Bone morphometry:** reconstruction was done using 3D Pro® after which VG studio Max® 3.0 was used for data analysis. The gray values representing the container were excluded using the import histogram. To ensure that only the bone material was being examined, manual surface determination was applied ascertaining that the background was removed without compromising the sample material of interest. The region of interest was then selected for surface determination.

**Data collection:** after selecting the distal epiphysis as a region of interest, the trabecular number, thickness, spaces and volume were obtained under “morphometrics” on VG studio. Bone length was obtained using the built-in caliper (Table 1).

**Sample size suitability and bias:** to determine the minimum number of pups required, sample size was estimated using the “resource equation” method as multiple endpoints were measured. This method measures the value “E”, which refers to the degrees of freedom of analysis of variance (ANOVA) and should be between 10 and 20 [9]. This value was calculated as follows: E = Total number of animals - total number of groups. Ethanol group E= (10x3)-3=27; saline groups: E= (10x3)-3=27; untreated group: E = (6x3)-3=15.

**Statistical analysis:** the data were managed in Microsoft Excel 365 (Microsoft Corporation) and analyzed using SPSS® version 28, IBM®, 2022. The saline and untreated groups saved as controls and were matched against the ethanol group which was exposed to the gestation alcohol. The untreated group controlled for confounding that could emanate from oral gavage. The Shapiro Wilks test was used to test for normality. Since the data were parametric, ANOVA with LSD post-hoc was used to test for group differences, with both the saline and untreated group compared again each other and against the ethanol group. Binary logistic regression was used to predict group membership into either the ethanol or saline control group and determine the parameters most affected by gestational alcohol to arrive at the observed outcome. Sensitivity was considered as the proportion of samples the regression model correctly classified into the correct grouping. The data are reported as mean ± standard deviation. Significance level was set at p < 0.05.

**Ethical considerations:** the study was approved by the Animal Ethics Committee, University of Witwatersrand (AESC 2015/27/15C) for use of animals.

## Results

### Rat participants

**Dams:** the plasma alcohol concentration was calculated from dams in the saline (n=5) and ethanol group (n=5). No measurements were taken from the untreated group.

**Bone samples:** since 2 pups were used as follows: ethanol group (n=10), saline controls (n=10) and untreated controls (n=6), this provided double the number of tibiae in each group. Therefore, the tibia used were as follows: ethanol group (n=20), saline controls (n=20) and untreated controls (n=12).

### Outcome data

**Blood alcohol concentration:** the plasma alcohol concentration for the saline control group was negative (or undetectable) whereas 81.53mg/dl was detected for the ethanol.

**Figure 2:**
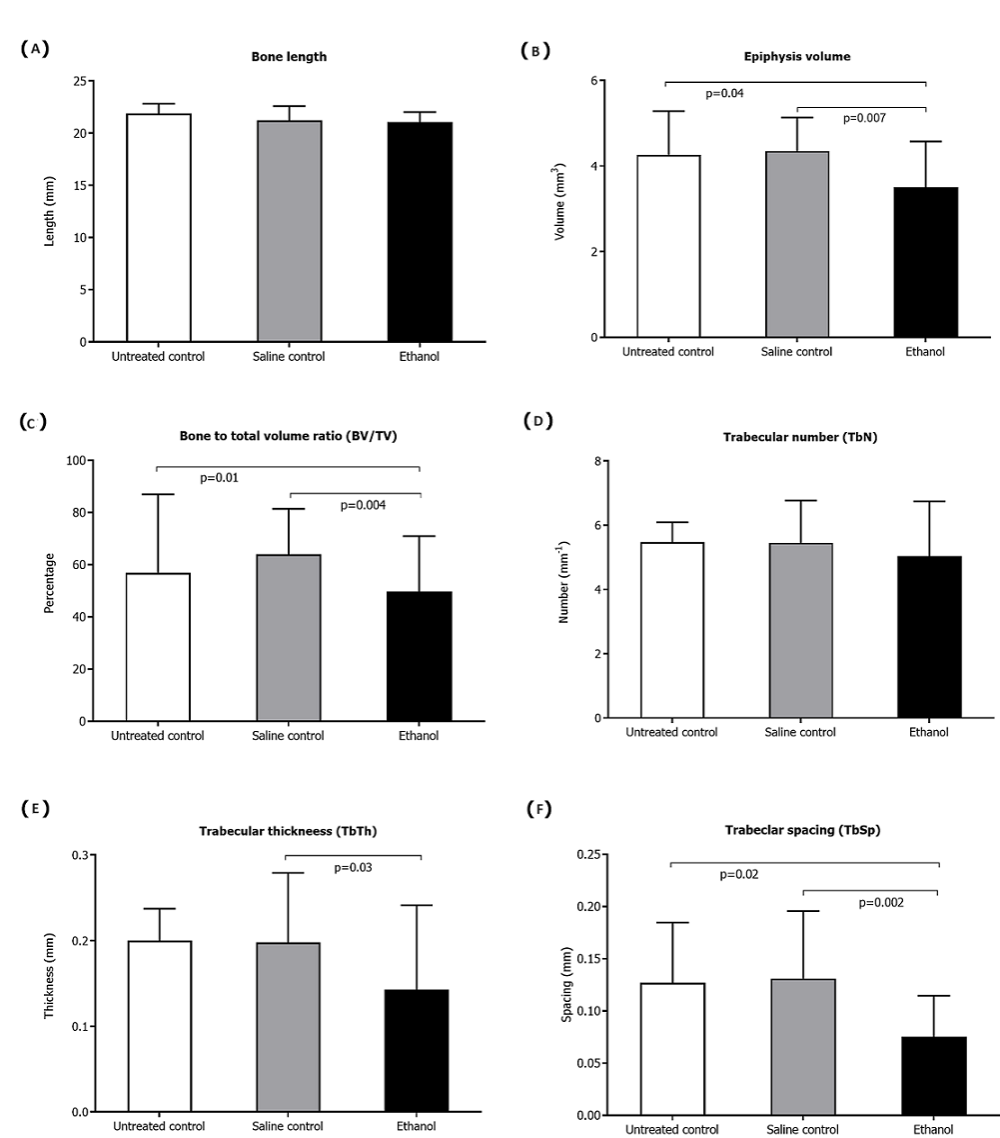
osteometric measurements and trabeculae morphometric parameters of the distal tibiae: A) full bone length; B) epicondylar breadth; represented as means for the saline control and ethanol group; C) bone to total volume (BV/TV); D) trabeculae thickness (TbTh); E) trabeculae number (TbN) and F) trabeculae spacing (TbSp) represented at the distal epiphysis of the tibia; error bars represent standard deviation

**Bone length distal metaphysis volume:** the mean full bone length was similar in all three groups studied (ethanol, mean=21.08mm ±0.95; saline control, mean=21.23mm ±1.38 and untreated control, mean=21.88mm ±0.94) (Figure 2A). In contrast, the distal metaphysis volume showed differences between the study groups, with the ethanol group showing a significantly smaller volume (Mean=3.51mm^3^±1.07) than the saline (Mean=4.35 mm^3^±0.78) and untreated controls (Mean=4.26 mm^3^±1.02) (p=0.007 and p=0.04 for the ethanol group compared to the saline and untreated groups respectively) (Figure 2B).

**Figure 3:**
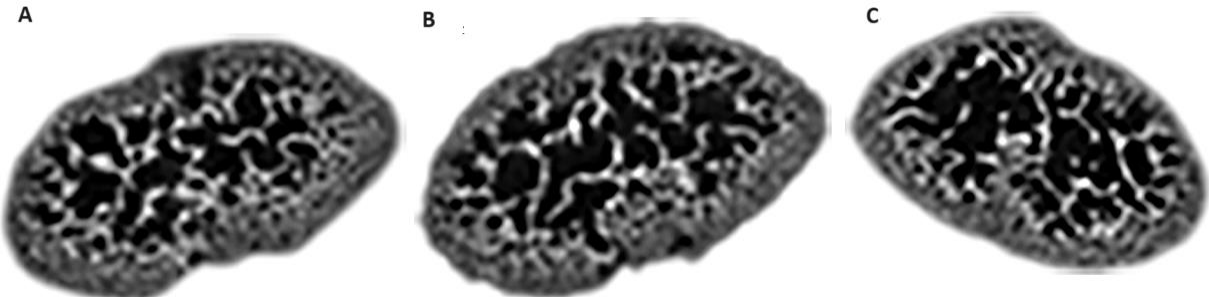
trabecular morphology; representative slices of: A) distal end in an untreated control showing more trabeculae and less spacing; B) distal end in a saline control showing moderate trabeculae and spacing; C) distal end of the ethanol group showing fewer trabeculae and wider spacing

**Trabecular morphometric parameters:** the ethanol group (Mean=54.73% ±14.53) had less bone to total volume (BT/TV) compared to the saline (Mean=66.81% ±11.05) and untreated controls (Mean=68.83% ±15.35). This difference was significant for the ethanol and saline groups (p<0.001) (Figure 2C). The ethanol group (Mean= 0.14 ±0.10) showed the thinnest trabeculae (TbTh) of all three groups in the study, being significantly lower than the saline controls (Mean= 0.20 ±0.08) (p=0.03) (Figure 2D). The number of trabecular (TbN) was similar in all three groups (Figure 2E) (ethanol, mean=5.04 ±1.70; saline control mean=5.46 ±1.32 and untreated control mean=5.48 ±0.61). However, differences in trabeculae spacing (TbSp) occurred (Figure 2F), with the ethanol group (Mean=0.08mm ±0.04) (Figure 3A) displaying less spacing than the saline (Mean=0.13mm ±0.06) (Figure 3B) and untreated group (mean=0.13mm ±0.06) (Figure 3C). These differences were statistically significant for both the ethanol and untreated controls (p=0.002 and p=0.02 for the ethanol group compared to the saline and untreated groups respectively).

**Trabecular morphometric parameters discriminating the ethanol and saline groups:** the effect of gestational ethanol on bone volume to total volume ratio, trabecular thickness, number, and separation was assessed using binary logistic regression in the ethanol and saline control groups as they had an adequate sample size for the analysis. The parameters used could reliably distinguish between the two groups. A test of the full model against a constant only model was statistically significant, indicating that the predictors as a set, reliably distinguished between ethanol or saline control group membership (Chi-square = 23.748, p < 0.001 with df = 4). Nagelkerke´s R^2^ of 0.577 indicated a moderately strong relationship between prediction and grouping. The Wald criterion demonstrated that only trabecular spacing (TbSp) predicted membership (Table 2). Prediction success overall was 78.6% (84.2% for the ethanol group and 73.9% saline controls) (Table 3).

**Table 2:** trabecular morphometric variables in the equation

Variables in the equation						
	**B**	**SE**	**Wald**	**df**	**Sig**	**Exp (B)**
BV/TV	10.212	6.094	2.808	1	0.094	27238.4
TbTh	7.822	10.542	0.551	1	0.458	2494.944
TbN	0.685	0.423	2.619	1	0.106	1.984
TbSp	41.665	16.795	6.155	1	0.013	12438520
Constant	-14.685	5.475	7.194	1	0.007	0

TbTh: trabeculae thickness; TbN: trabeculae number: TbSp: trabeculae spacing;BV/TV: bone to total volume

**Table 3:** group membership classification from trabecular morphometric parameters

Classification Table				
Observed		**Predicted**		
		**Group**		**Percentage correct**
		**Ethanol**	**Saline control**	
**Group**	Ethanol	16	3	84.2
	Saline control	6	17	73.9
Overall percentage				78.6

## Discussion

The current study employed micro focus X-ray computed tomography to understand how prenatal alcohol exposure affects bone development in the Sprague-Dawley rat tibia. This in-turn results in diminished stature of the offspring [10-12]. Tibia length was unchanged, but the volume of the distal metaphysis was smaller in the ethanol group suggesting a diminished size in this dimension of the tibia. Logistic regression showed that distal trabecular separation was the main parameter affected the most in gestational alcohol in the tibia. There was a negative correlation of trabecular thickness and spacing in the ethanol group.

**Osteometric and bone internal morphology:** the bone length of the tibia was similar among all three groups in the study. In contrast, previous studies found shorter tibiae in the ethanol group [1,13,14]. In the present study, we used 25.2% ethanol whereas other researchers used a higher ethanol concentration of 36% in studies [1,13,14]. The effects of prenatal ethanol exposure on bone are dose dependent as [1,14,15] showed that 36% ethanol has more adverse effects on bone than 25% and 15% ethanol administered in gestation. A possibility exists that we could have found shorter bone in the alcohol group had we used a higher alcohol percentage. Considering the relatively high rate of alcohol metabolism in rats, these animals in our study were only exposed to ethanol for approximately 2 hours per day as that is the time it takes for rodents to metabolise ethanol [5,14]. This, combined with the moderate dose of 25.2% ethanol could have possibly spared the animals from excessive harm. These results could potentially be different in humans as most people who drink during pregnancy are less educated and have lower socioeconomic status, which impacts their diet [8]. As the animals were on a nutritious balanced diet, this could have mitigated the effects of ethanol. Additionally, there was a 3-week postnatal period of no ethanol exposure which may have improved the chances of recovery in some respects such as bone length Streissguth *et al*.[16] and Spohr *et al*. [17] suggested that there is potential for catch up growth following prenatal exposure to ethanol. This proposition requires further studies to establish the facts.

**Trabecular bone:** in the present study, we found both lower bone volume to total volume (BV/TV) and trabecular thickness (Tb.Th) in the ethanol group in comparison with the controls. Having not seen differences in bone length among the three groups, we queried whether a size estimation method that includes more dimensions would yield the same observations. Linear measurements such as length are in a single dimension and as such tend to miss size information from other dimensions in three-dimensional space. We then, calculated the distal epiphyseal volume (part below the growth plate) as this is the portion rich in trabeculae. We found that the distal volume was significantly less in the ethanol group compared to the controls despite similar bone lengths. The trabecular number (TbN) was not affected in our study. This is similar to the findings of Maddalozzo *et al*. [18], who did not find any changes on the TbN in the distal tibial epiphysis. However, there was more trabecular spacing in the ethanol group. This, considered together with the lower BV/TV and TbTh in the ethanol group suggests that, although the bone length was similar in all groups, the internal morphology was not the same among the study groups. This means that prenatal alcohol exposure may affect internal architecture while sparing the external bone length. This disruption of the internal bone morphology may also explain why FAS children are prone to osteoporosis and fracture as they may have less bony material internally.

**Parameters most affected by prenatal ethanol exposure:** employing a binary logistic regression showed that the distal medullary canal area and trabecular spacing were the main parameters that determined group membership into either ethanol or saline control. This means that these two parameters (distal medullary canal area and trabecular spacing) are affected the most in gestational alcohol exposure. Our finding of a positive correlation between medullary canal area and cortical thickness indicates that the smaller medullary canal was coupled with thinner cortical bone. We propose that this may potentially explain weaker bones observed in FAS children. The negative correlation of trabecular thickness and spacing in the ethanol group indicates that thin trabecular occurred with more spacing in between. This would contribute to bone weakness. Our findings add light to the question of how osteoporosis and subsequent propensity to fracture may occur in prenatal alcohol exposure.

**Limitations:** the study was conducted in a laboratory and as such environmental factors were controlled. In contrast, life outside the laboratory such as human life may involve confounding environmental factors that may yield a different outcome. For example, physical activity may influence bone structural properties. A controlled alcohol dosage was administered; however, humans may drink variable amounts of alcohol. Fewer animals were used in the untreated group to reduce the number of animals required but equal samples would have minimized bias. As the untread group had fewer animals, we could only conduct binary logistic regression for the saline and ethanol group.

## Conclusion

Logistic regression showed that the distal trabecular separation was the main parameters affected the most in gestational alcohol in the tibia. The negative correlation of trabecular thickness and spacing in the ethanol group may be a contributor to bone weakness. These findings show that in-utero alcohol affects bone internal morphology in addition to the bone size Overall, this study supports the clinical observation of small stature in FAS children.

### What is known about this topic

- *There has been much research on the neurological anomalies and craniofacial dysmorphology in FASD with limited research on bone growth retardation;*
- *Most researches on FASD have focused on the effects of gestational alcohol exposure on fetuses with limited information on its effects on postnatal life*.

### What this study adds

- *This study adds information that intrauterine alcohol exposure disturbs internal microarchitecture resulting in lower bone volume to total volume (BV/TV) and trabecular thickness (Tb.Th) following prenatal alcohol exposure;*
- *These findings show that gestational alcohol exposure affects bone internal morphology in addition to the bone size. Overall, this study supports the findings of the clinical observation of small stature in FAS children*.
